# PARP1 in Carcinomas and PARP1 Inhibitors as Antineoplastic Drugs

**DOI:** 10.3390/ijms18102111

**Published:** 2017-10-08

**Authors:** Luyao Wang, Chao Liang, Fangfei Li, Daogang Guan, Xiaoqiu Wu, Xuekun Fu, Aiping Lu, Ge Zhang

**Affiliations:** 1 Law Sau Fai Institute for Advancing Translational Medicine in Bone and Joint Diseases, School of Chinese Medicine, Hong Kong Baptist University, Hong Kong, China; luyaoben@126.com (L.W.); liangchao512@163.com (C.L.); fayebalaba@live.com (F.L.); guandg0929@hotmail.com (D.G.); isawu199205@gmail.com (X.W.); ulric.fu@outlook.com (X.F.); 2 Institute of Integrated Bioinfomedicine and Translational Science, School of Chinese Medicine, Hong Kong Baptist University, Hong Kong, China; 3 Institute of Precision Medicine and Innovative Drug Discovery, School of Chinese Medicine, Hong Kong Baptist University, Hong Kong, China; 4 Shenzhen Lab of Combinatorial Compounds and Targeted Drug Delivery, HKBU Institute of Research and Continuing Education, Shenzhen 518000, China

**Keywords:** PARP1, carcinomas, PARP1 inhibitors

## Abstract

Poly (ADP-ribose) polymerase 1 (PARP1), the best-studied isoform of the nuclear enzyme PARP family, plays a pivotal role in cellular biological processes, such as DNA repair, gene transcription, and so on. PARP1 has been found to be overexpressed in various carcinomas. These all indicate the clinical potential of PARP1 as a therapeutic target of human malignancies. Additionally, multiple preclinical research studies and clinical trials demonstrate that inhibition of PARP1 can repress tumor growth and metastasis. Up until now, PARP1 inhibitors are clinically used not only for monotherapy to suppress various tumors, but also for adjuvant therapy, to maintain or enhance therapeutic effects of mature antineoplastic drugs, as well as protect patients from chemotherapy and surgery-induced injury. To supply a framework for understanding recent research progress of PARP1 in carcinomas, we review the structure, expression, functions, and mechanisms of PARP1, and summarize the clinically mature PARP1-related anticancer agents, to provide some ideas for the development of other promising PARP1 inhibitors in antineoplastic therapy.

## 1. Introduction

Poly (ADP-ribose) polymerase 1 (PARP1) is increasingly attractive as an anticancer therapeutic target in both preclinical studies and clinical trials. Many PARP1 inhibitors have been approved for treatment of human malignancies or under clinical investigation, such as olaparib (AZD2281), veliparib (ABT-888), and rucaparib (AG-014699, PF-01367338), for treatment of ovarian cancer, breast cancer, prostate cancer, pancreatic cancer, and unspecified solid tumors [1,2,3]. PARP1 has an essential role in cell proliferation, survival, and death, due to its effects on regulation of multiple biological processes [4,5]. A variety of tumor tissues have shown elevated expression of PARP1 protein, which may associate with deterioration, metastasis, and angiogenesis in tumors [6,7]. However, the role of PARP1 in various tumors remains obscure. In this paper, the structure, expression, and functions of PARP1 in cancers will be reviewed, followed by the presentation and analysis of some mature PARP1 inhibitors against human malignancies.

PARP1, the base excision repair (BER) protein, is widely known for its role in sensing damaged DNA and catalyzing DNA repair [8,9]. Initial studies report that PARP1 is a promising target for treatment of BRCA-deficient carcinomas. BRCA-mutant carcinomas, which are homologous recombination (HR) deficient and rely on PARP1-BER for survival, are highly sensitive to PARP1 inhibitors [10,11]. Array data also demonstrates good tumoricidal efficiency of PARP1 inhibitors on these DNA repair defective carcinomas [5,12,13].

As PARP1 research goes more in-depth, functions and mechanisms of PARP1 have been found to be more complicated, and even double-faced. Furthermore, many PARP1 inhibitors were demonstrated to be not only effective against familial DNA repair defective carcinomas, but also against HR-proficient cancers, like HR-proficient HER2^+^ breast carcinoma and Ewing’s sarcoma, in pre-clinical or clinical studies [14,15,16]. Currently, design of PARP1 inhibitors and PARP1 inhibitors under clinical trial receive considerable attention [17,18]. In this paper, we will not only review PARP1 inhibitors, but also introduce the role of PARP1 in DNA repair, gene transcription, inflammation, cell cycling, and angiogenesis during tumor development.

## 2. PARP1 Structure and Expression in Carcinomas

**Figure 1:**
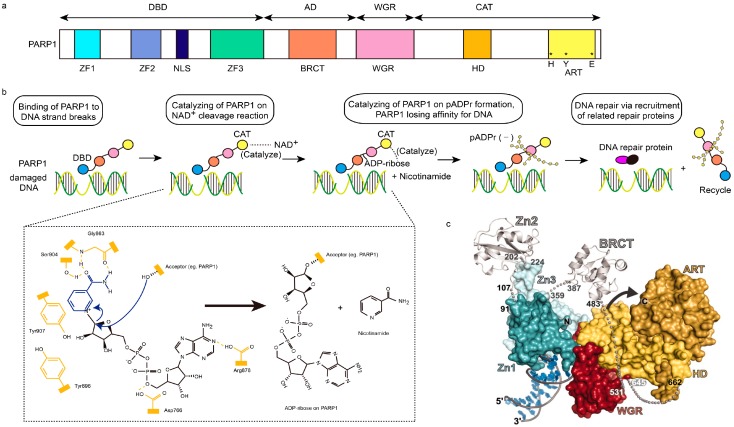
Structural characteristics of PARP1 and PARP1-based DNA repair. (**a**) Schematic representation of human PARP1 molecular architecture; (**b**) Structural process of PARP1-mediated DNA repair. After DNA-binding domain (DBD, blue ball) of PARP1 senses and binds damaged DNA, the NAD^+^ will be cleaved into ADP-ribose and nicotinamide, which are catalyzed by the catalytic domain (CAT, yellow ball) of PARP1. Then, poly (ADP-ribose) (pADPr) is synthesized on the acceptor PARP1 through the combination of ADP-ribose, assisted by the catalysis of CAT too. Subsequently, PARP1 leaves damaged DNA, due to the dense negative charge of pADPr, allowing the recruitment of related repair proteins and replication. In chemical formulae, domains in yellow represents active domains of PARP1, while blue and black parts represent NAD^+^. Hydroxyl of Ser904 and carbonyl and NH group of Gly863 in PARP1 interact with the amide moiety of NAD^+^ via hydrogen bonding interaction, while Tyr907 of PARP1 via π–π stacking interaction. The curved arrows in blue represent the nucleophilic attack by the 2′-hydroxyl of the acceptor site in PARP1, and the release of the nicotinamide procedures; AD, orange ball; WGR, red ball. (**c**) The PARP1/damaged DNA crystal structure. Zn1, dark green; Zn2, silver (left); Zn3, light green; BRCT, silver (right); ART, dark yellow; HD, light yellow; red, WGR [4,18,19].

PARP1 consists of four functional domains: the amino (N)-terminal DNA-binding domain (DBD), auto-modification domain (AD), WGR domain, and the carboxy (C)-terminal catalytic domain (CAT) [4,19]. DBD comprises two zinc fingers (ZFs: ZF1 and ZF2) for binding of PARP1 to single- and double-strand DNA breaks (SSBs, DSBs), and a nuclear localization signal (NLS), which is a DNA-nick sensor [20]. There is a third ZF (ZF3) which has been found exclusively in PARP1, rather than other PARP isoforms. It may be dispensable for DNA-binding, but plays an important role in PARP1 catalytic activity via relaying of “PARP1-DNA binding” signal to the catalytic domain [18,21]. AD is a central regulating domain, mainly containing a breast-cancer-susceptibility protein-carboxy terminus (BRCT) motif for auto-ADP ribosylation and mediating PARP1–protein interaction [4,22]. WGR, defined in accordance with its conserved Trp, Gly, and Arg residues, is an essential domain whose function remains unclear [19]. CAT consists of a helical subdomain (HD) and a conserved ADP-ribosyl transferase subdomain (ART). ART is composed of vital histidine (H) and tyrosine (Y) residues for NAD^+^ binding, and a glutamic acid (E) residue for polymerase activity [19,21,23,24] (Figure 1a).

PARP1, a nuclear enzyme, has now been identified in both the nucleus and cytoplasm of cancer cells. Investigation of PARP1 expression level in various tumors is essential to evaluate potential therapeutic effects, and side effects of PAPR1 inhibitors on each subtype of carcinomas.

A multitude of tumors have been found to be related with upregulated PARP1, when compared to adjacent normal tissues. Ossovskaya’s group investigated more than 8000 surgical tissue samples from malignant patients and healthy individuals. Results showed that PARP1 is overexpressed significantly in malignant tissues of BRCA-mutant, triple negative (TN) and receptor-positive breast carcinoma (BRCA-mutant/triple negative (TN) > receptor-positive), as well as uterine carcinoma, ovarian carcinoma, lung carcinoma, skin carcinoma, and non-Hodgkin’s lymphoma [25]. Of these, breast cancer has attracted the most attention. Clinical data of Domagala’s group showed that nuclear PARP1 expression was upregulated in most breast tumors, while the overexpression of nuclear–cytoplasmic PARP1 was present only in a small percentage of breast tumors [26]. Additionally, nuclear and nuclear–cytoplasmic PARP1 expressions were clinically found to be related to undesirable poorer prognosis and shorter overall survival in lymph node-negative early breast carcinoma [27,28]. Compared to non-small cell lung carcinoma (NSCLC), PARP1 protein level was higher in small cell lung cancer (SCLC), leading to higher sensitivity of SCLC to PARP1 inhibitors [29]. Brenner and members found that EWS-FLI1 genes in Ewing’s sarcoma could maintain PARP1 expression via a positive feedback loop [15]. Thus, Ewing’s sarcoma was significantly sensitive to PARP1 inhibitors. Galia’s group identified the upregulation of PARP1 expression in cellular nuclear compartments of glioblastoma multiforme, but not in the cytoplasm, via immunohistochemistry [30]. There were also other studies that demonstrated the overexpression of PARP1 in prostate carcinoma, colorectal carcinoma, pediatric central nervous system carcinomas, and testicular germ cell tumors, respectively [31,32,33,34] (Table 1). The above studies suggest that the expression level of PARP1 in cancer cells should be related to the sensitivity of various carcinomas to PARP1 inhibitors.

## 3. PARP1 Function and Mechanism in Carcinomas

### 3.1. Multifaceted Function on DNA Repair

**Figure 2:**
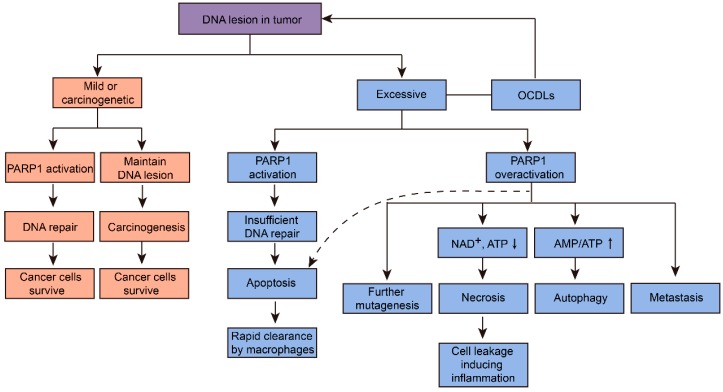
Cancer cells fate induced by different levels of PARP1-related DNA repair in tumor. PARP1 regulated various pathways due to the degree of DNA lesion in tumor. To survive, cancer cells will activate PARP1 to repair mildly damaged DNA, and harbor carcinogenetic genetic mutations via maintaining DNA lesions. In case of excessive DNA lesions, normal PARP1 activation will cause insufficient DNA repair, subsequently leading to cancer cell apoptosis. This may be a more desirable result of cancer cells in anticancer therapy, because apoptosis cells will then be cleared by macrophages rapidly. On the other hand, overactivation of PARP1 will lead to further mutagenesis, metastasis, and energy-depleted necrosis and autophagy of tumors. Necrosis will then further induce inflammation, which is another important carcinogenetic factor. Inhibition of PARP1 overactivation by PARP1 inhibitors will transform cells’ fate to apoptosis, subsequently reduce further mutagenesis, metastasis, autophagy, necrosis, and tumor-promoting inflammation (dashed arrow) [4,14,18].

PARP1-assisted DNA repair in cancers is complicated [4,14]. On one hand, tumor cells always harbor DNA repair defects to maintain the DNA lesions that can foster carcinogenesis. Additionally, damaging DNA of cancer cells is always used as the treatment mechanism of some anticancer agents. PARP1 inhibitors can further suppress the DNA repair process and drive cancer cell death, subsequently inhibiting cancers independently, or as anticancer assistant agents. On the other hand, DNA lesion in cells induced by excessive reactive oxygen species (ROS) is one of the major factors of carcinogenesis. In microenvironment homeostasis of cellular, this can be averted by PARP1-BER pathway. However, oxidative clustered DNA lesions (OCDLs), which mean excessive DNA lesions occurred frequently in tumors, may disturb the microenvironment homeostasis and lead to more severe DNA insult. Sustained activation of PARP1 and insufficient DNA repair will lead to further mutagenesis, metastasis, and energy-depleted necrosis of tumors. In this condition, PARP1 inhibitors could suppress mutagenesis and metastasis, as well as turn necrosis to apoptosis aiming to avoid inflammation-mediated extra cytotoxicity (Figure 2).

PARP1-assisted DNA repair is a nicotinamide adenine dinucleotide (NAD^+^)-related energy consuming process. After DNA-binding domain (DBD) of PARP1 binding to the damaged DNA, the NAD^+^ will be cleaved into ADP-ribose and nicotinamide, which is catalyzed by the catalytic domain (CAT) of PARP1. Then poly (ADP-ribose) (pADPr) is synthesized on acceptor proteins (e.g., PARP1, histones, or transcription factors) through the combination of ADP-ribose, also assisted by the catalysis of CAT. Subsequently, PARP1 leaves damaged DNA, owing to the dense negative charge of pADPr, allowing the recruitment of related repair proteins and replication [35] (Figure 1b). This process downregulates NAD^+^ and ATP levels in the cell, which can be recycled in a normal microenvironment [18]. However, over-activation of PARP1-related DNA repair will disturb the energetic balance in the cell, leading to rapid energy depletion, and then, cell necrosis [14,18]. Inhibition of PARP1 prevents the exhaustion of ATP in cells, subsequently minimizing related side effects resulting from chemotherapy and surgery-induced injury.

Nowadays, a progressively increasing number of studies focus on investigating the structural aspect of the mechanism of PARP1-related DNA repair. The X-ray co-crystal structure between PARP1 and NAD^+^ revealed the extensive hydrogen bonding interactions between the amide moiety of NAD^+^ with the hydroxyl of Ser904, and carbonyl and NH group of Gly863. Additionally, other interactions in the network of PARP1 and NAD^+^, such as π–π stacking interactions between the amide moiety of NAD^+^ and Tyr907 of PARP1, were also discovered [18] (Figure 1b). Langelier and members provided the X-ray crystal structure to illustrate the binding mode of human PARP1 (ZF1, ZF3, and WGR-CAT) with DNA DSBs, and advised that the upregulation of CAT domain dynamics is essential to DNA-dependent activation of PARP1 [19] (Figure 1c). Recent study of David Neuhaus’ research group [24] demonstrated the complexity of ZF1–ZF2 domain, with dumbbell DNA as the minimal structural unit for first-stage interaction between PARP1 and SSBs. The binding ability of ZF1–ZF2 domain to SSBs is slightly less strong than PARP1. In this study, NMR/X-ray hybrid structure of ZF1–ZF2 bound to an SSB was presented, which showed how two flexibly linked zinc fingers of PARP1 bound to the break of damaged DNA. Nuclear Overhauser effect (NOE) contacts within ZF1–ZF2, ZF1-5′ stem/T23 of DNA, and ZF2-3′ stem of DNA indicated the interaction interfaces of domain–domain and protein–DNA. Gibson’s group identified two gatekeeper residues of PARP1, and depicted the structural interaction between gatekeeper residues and NAD^+^. They also demonstrated that inhibition of PARP1 could promote the pausing of RNA polymerase II, which is another key factor of carcinomas [36].

### 3.2. PARP1 in Gene Transcription

PARP1 associates with DNA through binding or interacting with nucleosomes, and other chromatin-related proteins, including gene transcription factors, transcription machinery, and chromatin modulators [23,37]. Dysregulation of gene transcription has been clinically demonstrated as a vital influencing factor of cancer cell proliferation, invasion, metastasis, and response to therapy. PARP1 is carcinogenic in tumors with and without DNA lesions, due to its functions in both DNA repair and transcriptional regulation, positively or negatively. The activation function of PARP1 on the transcription of many oncogenes, such as vascular endothelial growth factor receptor 1 (VEGFR1) gene and hypoxia-inducible factor 1α and 2A (HIP1α and HIF2A) genes, was tumorigenic [5,38,39]. For instance, PARP1, supporting the transcription of androgen receptor in androgen receptor-positive prostate carcinoma, is necessary for tumor cell proliferation [31]. Recent research demonstrated that PARP1 would induce transcriptional activation of the melanocyte-lineage survival oncogene (MITF), indicating its role in melanomagenesis [40]. Similar effect exists were observed when PARP1 binds to transcription factors [5]. For example, the binding of PARP1 to NF-κB could activate the expression of melanoma growth stimulatory activity-regulated protein (CXCL1), subsequently improving the progression of melanoma [41]. Thus, inhibition of PARP1 may be associated with transcriptional silencing of oncogenes. PARP1 does not always modulate transcription of genes positively. In malignancies, downregulating transcription of tumor suppressors by PARP1 can also promote tumor growth and progression [42]. The repressive effects of PARP1 on classical tumor suppressing agents p53 and APC, in cancers, are good examples [43]. Accordingly, PARP1 inhibitors may inhibit tumor progression via recovering the tumor suppressing function of related factors.

**Figure 3:**
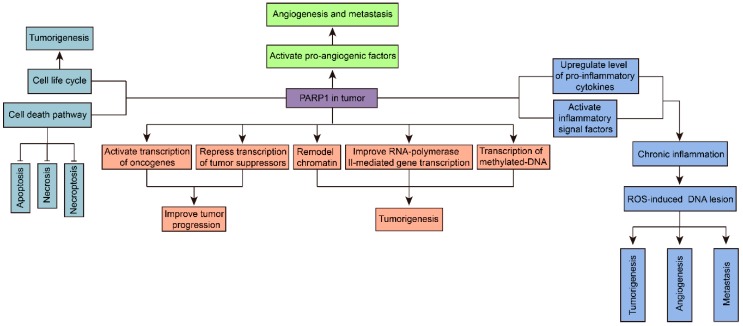
The multifunction of PARP1 in tumorigenesis. PARP1 is considered to promote tumor development potentially through many pathways. Briefly, PARP1 regulates gene transcription through interacting with transcription factors, transcription machinery, and chromatin modulators. Hyperactivated PARP1 upregulates inflammatory signal factors in tumors. Also, PARP1 modulates cancer cellular life cycle via regulating cellular mitosis and cell death pathways, including apoptosis, necrosis, and necroptosis. PARP1 activates pro-angiogenic factors and induces angiogenesis and metastasis in tumors.

PARP1 also plays a significant role in maintaining integrity of genes [37]. Besides the transcription modulation pathway above, PARP1 also regulates gene expression through chromatin remodeling, RNA polymerase II, and DNA methylation pathways [44,45,46]. The functional study of PARP1 on chromatin modulation has lasted for several decades, since 1999 [47]. In general, PARP1 regulates chromatin structure through modulating the activity and location of histone in chromatin [42,48]. The binding of PARP1 and nucleosome can modulate chromatin to a super-nucleosomal structure which suppresses transcription [48]. PARP1 can also disassociate linker histone (H1), subsequently improving RNA polymerase II-mediated gene transcription [49]. DNA methylation is also a common reason for tumorigenesis in various carcinomas, similar to gene mutation. PARP1 inhibition can downregulate the transcription of methylated DNA [50]. PARP1-mediated modulation of gene transcription has been demonstrated to play some vital roles in suppressing tumors. However, the exact effects of PARP1 on transcription and DNA methylation pathways at specific genes are complicated and obscure, indicating the manipulation of gene transcription via PARP-1 in tumors is still too immature and tender for clinical cancer therapy (Figure 3).

### 3.3. PARP1 in Tumor-Promoting Inflammation

Chronic inflammation, which will result in ROS-induced DNA lesion, may be another major factor of carcinogenesis. Hyperactivated PARP1 upregulates inflammatory signal factors like NF-κB, subsequently leading to inflammation and further tumorigenesis [14]. NF-κB, the classical tumor-promoting inflammatory signal, is a class of transcriptional factor which can activate pro-inflammatory transcription machinery. P65 (RelA), p50, p52, Rel B, and c-Rel all belong to NF-κB family [51]. Additionally, the interaction between NF-κB and PARP1 upregulates the level of pro-inflammatory cytokines like tumor necrosis factor α (TNFα) and interleukin 6 (IL6), which will also initiate tumor-promoting inflammation [52].

On the other hand, NF-κB-mediated chronic inflammation facilitates tumors to some malignant phenotypes which can escape from immune surveillance [53]. NF-κB also plays a significant role in carcinoma progression, metastasis, and angiogenesis [54]. Thus, inhibition of overexpressed PARP1 in human malignances may work on repressing chronic inflammatory-induced cancer progression, through blocking the NF-κB activation effect of PARP1. In addition, studies found that NF-κB was hyperactivated in HER2^+^ breast carcinoma, and could block cancer cells apoptosis [55]. Interestingly, another study demonstrated that HER2^+^ breast carcinoma was highly sensitive to PARP1 inhibitors, due to the suppressing effect of PARP1 inhibitors on NF-κB inflammatory signal [16]. This is a good supporting instance for the theory that PARP1 inhibition is also effective in DNA repair proficient carcinomas. PARP1 inhibitors can not only reduce inflammation-related side effects caused by chemotherapy and surgery injury, but also be used as potential preventative and antitumoral agents against carcinomas.

Besides the NF-κB-mediated inflammatory mechanisms, inflammation can induce cancer in other pathways. In chronic inflammatory microenvironment, upregulation of PARP1 will lead to an increase of iNOS, which is an integral unit of ROS/iNOS, subsequently inducing cellular DNA lesions. Thus, PARP1 inhibitors also serve as cell protectors against enhanced ROS/iNOS and inflammation-induced carcinogenesis (Figure 3) [56].

### 3.4. PARP1 in Cell Cycling Regulation

Carcinomas are characterized by slow metabolism, a low cell death rate, and high proliferation rate. Weakened mitosis, downregulation of cell death factors, evasion of growth repressing factors, or upregulation of anti-death factors may all act as tumor drivers. PARP1 modulates the cancer cellular life cycle via regulating cellular mitosis and cell death pathways, including apoptosis, necrosis, and necroptosis [5]. Mitosis is important for appropriate chromosome segregation. Cells with dysregulated mitosis survive, but cannot conduct appropriate chromosome segregation, subsequently leading to genomic mutants, and even tumorigenesis. This may also be one of the reasons for tumor deterioration [57].

PARP1 also modulates cancer cell cycle via controlling cell death (Figure 3). Initially, PARP1 in cancer cells will activate the apoptosis pathway. Nevertheless, when DNA damage is severe, overactivated PARP1 will lead to exhausting NAD^+^/ATP-induced necrosis, as described above. Accordingly, inhibition of PARP1 preserves energy, and keeps PARP1 at relatively normal levels, subsequently causing the generation of apoptosis cancer cells which can be cleared by macrophages [4]. In addition, PARP1 also regulates necroptosis of cancer cells through extracellular signal-regulated kinases (ERK), and C-jun N-terminal kinase (JNK) pathways. ERK, which can improve anti-apoptotic protein expression and inhibit activity of the caspase family (apoptosis-inducing enzymes), serves to protect cancer cells from death [58]. PARP1 can stimulate ERK, inducing survival of malignant cells. JNK, which activates PARP1 continuously, will lead to NAD^+^/ATP depletion-induced necrosis. Interestingly, PARP1 can also simulate JNK, which makes a JNK–PARP1–JNK sustained activating loop and further drives nonapoptotic cell death [59]. PARP1 inhibition, which blocks the JNK–PARP1–JNK loop and ERK-mediated anti-apoptotic protein expression, will result in cancer apoptosis. In general, downregulation of PARP1 could modulate many factors in the cell life cycle and cell death to suppress proliferation of carcinomas, which has been demonstrated in PARP^−/−^ mice models and human breast cancer cells, and so on [49,60].

### 3.5. PARP1 in Metastasis and Angiogenesis

Multiple research studies have demonstrated the anti-angiogenesis and even anti-metastasis effect of PARP1 inhibitors. ERK, which can inhibit cancer cell apoptosis as mentioned above, was also found to be highly related to tumor metastasis and angiogenesis. Stimulation of ERK by PARP1 will improve the activity of pro-angiogenic factors, such as vascular endothelial growth factor (VEGF), platelet/endothelial cell adhesion molecule (PECAM1/CD31), transmembrane signal protein syndecan-4 (SDC-4), and hypoxia inducible factor (HIF), inducing angiogenesis and metastasis [61,62]. In addition, immunohistochemistry studies on clinical human epithelial ovarian tumor samples found that PARP1 was significantly associated with microvascular density (MVD, CD34), tumor volume, and lymphatic metastasis. In vitro research demonstrated that PARP1 inhibition could repress related human umbilical vein endothelial cell (HUVEC) tubule formation [63]. The study also found that PARP1 was positively related to vimentin (angiogenic intermediary filament) of endothelial cells in malignancies. Inhibition of PARP1 reduced vasculogenic mimicry in melanoma cells, indicating the potential of PARP1 inhibitors on attenuating tumor metastasis [64]. In addition, as mentioned above, the classic tumor-promoting inflammatory factor NF-κB is significantly associated with carcinomas angiogenesis and metastasis. Metastasis may also link to PARP1-mediated chronic inflammation in carcinomas (Figure 3).

Furthermore, PARP1-mediated carcinoma metastasis may be in gene transcription manner. For instance, Choi’s research demonstrated that inhibition of PARP1 blocked lung carcinoma metastasis in a DNA repair-independent mechanism. PARP1 upregulates metastasis of lung cancers by improving PARP1-mediated transcription of S1000A4 and CLDN7. Besides, it also promotes metastasis of lung carcinoma in brain and bone via facilitating cancer cell extravasation, invasion, and self-renewal, resisting apoptosis, as well as changing the brain microenvironment to induce relapse of lung carcinoma cells to the brain [65].

## 4. PARP1 Inhibitors against Carcinomas

PARP1 inhibitors, as used in monotherapy or maintenance therapy, have clinically presented promising efficiency against ovarian, breast, and other carcinomas [66,67]. Up until now, preclinically studied PARP1 inhibition methodologies are divided into two types: pharmacological inhibition, and genetic knockdown. PARP1 inhibitors include chemical compounds and nucleic acids [65]. In general, the antineoplastic mechanism of currently mature PARP1 inhibitors mainly focuses on DNA repair pathways [67,68].

The earliest discovered PARP1 inhibitors included nicotinamide/ benzamide derivatives, which repress PARP1-induced DNA repair and improve the sensitivity of cancer cells to DNA damaging agents [69,70,71]. Nicotinamide, the catabolite of NAD^+^ in PARP1-mediated DNA repair pathway, has become the investigating structural model for PARP1 inhibitors. Relevant PARP1 inhibitors have entered late-stage clinical trials or been approved as anticancer drugs, working with other anticancer chemotherapy drugs or being used for monotherapy. Thereinto, olaparib (AZD2281, trade name: Lynparza, given orally), the first single drug for PARP1-mediated carcinoma therapy, clinically showed great inhibition ability against BRCA-mutation carcinomas [72]. Its indications included ovarian carcinoma, breast carcinoma, pancreatic carcinoma, prostate carcinoma, and solid tumors like Ewing’s sarcoma, and so on [1,66] (Table 2). In 2014, the European Medicines Agency (EMA) approved olaparib as a maintenance therapeutic drug for patients with BRCA-mutation ovarian carcinoma following platinum chemotherapy. The United States Food and Drug Administration (FDA) also approved olaparib as a PARP1/2-related drug against BRCA-defective ovarian carcinoma with three or more pro-chemotherapies [73]. Preclinical and clinical investigations of olaparib against other malignancies are in progress. However, as the substrate of the p-glycoprotein efflux pump, olaparib might be resisted by some patients [74]. Studies initially discovered that some subtypes of breast cancer might be resistant to olaparib [75]. In addition, side effects, including bone marrow problems called myelodysplastic syndrome (MDS) or acute myeloid leukemia (AML), are nonnegligible.

Following olaparib, an array of PARP1-based antitumor agents are being discovered, have entered clinical trials, or have been approved to be used as anticancer drugs. Niraparib (MK-4827, given orally) is another well investigated PARP1 inhibitor approved by the FDA as a maintenance therapy drug with platinum-sensitive recurrent ovarian carcinoma [76]. Other adaptation diseases also include breast carcinoma, melanoma, and unspecified solid tumors [77]. Rucaparib (AG-014669, PF-01367338, trade name: Rubraca, given orally), an FDA granted and recently approved PARP1-based drug for BRCA-mutant ovarian carcinoma patients, is indicated for therapy one line earlier than olaparib [78]. It has also been investigated against breast, pancreatic, fallopian tube, and peritoneal carcinomas [79]. Further investigations have significantly found the tumoricidal effect of rucaparib in human non-BRCA-mutant ovarian cancer for both monotherapy and combination therapy [78,80]. Veliparib (ABT-888), a benzimidazole-4-carboxamide derivative, has been preclinically studied against breast, ovarian, lung, pancreatic, prostate, testicular and colorectal cancers, and other unspecified solid tumors. Most of clinical studies of veliparib were for combination therapy (www.clinicaltrials.gov) [81,82,83]. Talazoparib (BMN-673) has been reported to be used as both a mono-therapeutic and combination therapeutic drug with leukemia, Ewing’s sarcoma, BRCA-mutant and proficient ovarian carcinoma, breast carcinoma, and osteosarcoma [84,85,86,87]. It is a promising PARP1-based anticancer drug candidate, because its PARP–DNA complex trapping ability is almost 100-fold stronger than that of olaparib and rucaparib [88] (Table 2). Apart from these well-known PARP1 inhibitors, other novel compounds also demonstrate potential for PARP1 inhibition before clinical trials. For example, one natural product, ZINC67913374, is more stable than olaparib when interacting with PARP1 in molecular dynamics simulations [89]. With pharmacophore and co-crystallization studies, a novel PARP1 inhibitor OL-1 was found to induce cell death and inhibit cell migration in BRAC1 mutant MDA-MB-436 cells [90].

General well-investigated eligibilities of PARP1 inhibitors include ovarian and breast carcinomas, both BRCA-mutant and BRCA-proficient. Additionally, most of the currently discovered PARP1 drugs are taken orally, which could be one of the advantages of PARP1 drugs, offering patients a more comfortable and acceptable therapeutic experience. Due to the DNA repair-related tumoricidal mechanism of currently mature PARP1 anticancer drugs, reduction in pADPr formation has generally been used to evaluate therapeutic efficiency of PARP1 inhibitors as pharmacodynamic (PD) biomarkers. However, clinical results showed that there was no obvious correlation between the clinical tumoricidal efficiency and pADPr-related PD read-out for some PARP1 inhibiting drugs, such as olaparib and rucaparib. As poly (ADPr) is generated not only by PARP1, but also by other PARP isoforms, we speculate that this could be attributed to the selective inhibitory potency of the drugs on PARP1/2, rather than other PARP isoforms. Thus, other PD biomarkers, including the one which indicates PARP1 expression level and basic activity, as well as genomic approaches, should be taken into account in predicting or evaluating the sensitivity of PARP1 inhibitors [91,92].

From the perspective of the structural angle, present mature PARP1 inhibitors entered clinical trials always contain nicotinamide-mimic motifs to inhibit PARP1/NAD^+^ interaction via competing with the nicotinamide pocket of NAD^+^ [93]. All the PARP1-inhibiting drugs in clinical use contain a benzamide unit in a ring. This ring is essential for activity of PARP1 inhibitors because it holds the amide in the plane of the benzene ring. In olaparib, rucaparib, and talazoparib, this amide-containing ring is covalent. In veliparib and niraparib, the ring is formed from five covalent bonds and a strong intramolecular hydrogen bond, which holds the conformation of the pharmacophore. Nicotinamide/benzamide-mimic pharmacophores have become a classic structural basis for investigating and designing PARP1 inhibitors. However, due to the structural characteristic, many mature PARP1 inhibitors should have the potential to block most of PARP family enzymes which can interact with NAD^+^ [94]. Nowadays, more and more novel chemical compounds or nucleic acids, which do not imitate nicotinamide and may be PARP1-specific inhibitors, are gradually emerging. Computational methodology, which screens libraries of chemical compounds, proteins, and nucleic acids to find key features of interactions between drug candidates and targets, has already become a vital tool for PARP1-based drug discovery [95].

## 5. Conclusions and Future Perspectives

In this paper, the structure, expression, and functions of PARP1 in cancers were reviewed. PARP1 has been preclinically or clinically found to be overexpressed in various carcinomas, such as various subtypes of breast cancers, ovarian cancer, and lung cancer. Furthermore, the expression level of PARP1 in these cancers is different. This may be related to the sensitivity of carcinomas to PARP1 inhibitors. This should be considered when precisely choosing therapeutic strategies for patients with carcinomas. Besides recognizing lesion DNA and catalyzing DNA repair, PAPR1 also regulates cancer cells via modulating other cancer-related biological processes, including gene transcription, inflammation, cell life cycle, angiogenesis, and so on. Due to its multiple functions, PARP1 inhibitors can not only cure BRCA-deficient cancers, but also is efficient for BRCA-proficient cancers. PARP1 inhibitors are reported to be effective on blocking cancer metastasis.

As mono-therapeutic or combination therapeutic drugs, the mature PARP1 inhibitors against human malignancies were overviewed. Following the FDA-approved drugs olaparib, niraparib, and rucaparib, other well-investigated PARP1 inhibitors such as veliparib and talazoparib have entered clinical trials. The general well-investigated eligibilities of these PARP1 inhibiting drugs included ovarian and breast carcinomas, both of which presented high sensitivity to these drugs. Currently, mature PARP1 inhibitors mainly focus on DNA repair pathways. From the structural angle, all these PARP1 inhibitors contain amide motif as an active group. Investigating new PARP1-based antineoplastic drugs according to this rule may be easy. However, this also limits the development of PARP1 inhibitors. Thus, further studies on the expression level and location of PARP1 in numerous cancers to assist to find more eligibilities of PARP1 inhibitors may be significant. Investigating and designing chemical compounds and nucleic acids, which can inhibit tumor progression and metastasis based on other PARP1-related functions instead of DNA repair pathways, may expand the indications range of PARP1 inhibitors, and be helpful to break the bottleneck in investigating novel PARP1 drugs.

## Figures and Tables

**Table 1:** Expression level of PARP1 in different carcinomas.

Cancer Types	Expression of PARP1
Breast Cancer	Up-regulated
Ovarian Cancer	Up-regulated
Uterine Cancer	Up-regulated
Lung Cancer	Up-regulated
Skin Cancer	Up-regulated
Non-Hodgkin’s Lymphoma	Up-regulated
Glioblastoma Multiforme	Up-regulated
Prostate Cancer	Up-regulated
Ewing’s Sarcoma	Up-regulated
Colorectal Cancer	Up-regulated
Pediatric Central Nervous System Cancer	Up-regulated
Testicular Germ Cell Tumor	Up-regulated

**Table 2:** Structure and indications of mature anticancer PARP1-based drugs entered clinical trials.

Drug	Structure	Active Structural Group	Biophysical Parameters (PARP1)	Well Studied Cancer Types	Typical Clinical Trials No. (The Latest Stage)	Phase	REF
Olaparib (AZD2281)	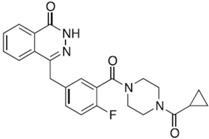	Amide motif enclosed in cyclic ring	IC_50_ = 5 nM	Ovarian carcinoma	NCT02476968 (Phase 4)	1–4	[1,73]
				Breast carcinoma	NCT02032823 (Phase 3)	1–3	
				Prostate carcinoma	NCT02987543 (Phase 3)	1–3	
				Pancreatic carcinoma	NCT02184195 (Phase 3)	1–3	
				Ewing’s sarcoma	NCT01583543 (Phase 2)	1–2	
				Gastric carcinoma	NCT01924533 (Phase 3)	1–3	
				Lung carcinoma	NCT03009682 (Phase 2)	1–2	
				Germ Cell Tumor	NCT02533765 (Phase 2)	1–2	
				Colorectal carcinoma	NCT00912743 (Phase 2)	1–2	
				Unknown solid tumors	NCT03233204 (Phase 2)	1–2	
Niraparib (MK-4827)	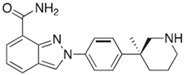	Conventional embedded primary amide	IC_50_ = 3.2 nM	Ovarian carcinoma	NCT01847274 (Phase 3)	1–3	[76,93]
				Breast carcinoma	NCT01905592 (Phase 3)	1–3	
				Endometrial carcinoma	NCT03016338 (Phase 2)	1–2	
				Uveal melanoma	NCT03207347 (Phase 2)	1–2	
				Fallopian tube carcinoma	NCT02657889 (Phase 2)	1–2	
				Peritoneal carcinoma	NCT02657889 (Phase 2)	1–2	
Rucaparib (AG-014669, PF-01367338)	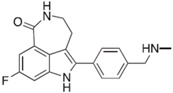	Amide motif enclosed in cyclic ring	IC_50_ = 1.4 nM	Ovarian carcinoma	NCT02855944 (Phase 3)	1–3	[78,80,96]
				Fallopian tube carcinoma	NCT02855944 (Phase 3)	1–3	
				Peritoneal carcinoma	NCT01968213 (Phase 3)	1–3	
				Prostate carcinoma	NCT02975934 (Phase 3)	1–3	
				Pancreatic carcinoma	NCT02042378 (Phase 2)	1–2	
				Breast carcinoma	NCT02505048 (Phase 2)	1–2	
Veliparib (ABT-888)	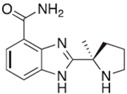	Conventional embedded primary amide	K_i_ = 5.2 nM	Breast carcinoma	NCT02032277 (Phase 3)	1–3	[82,97,98]
				Ovarian carcinoma	NCT02470585 (Phase 3)	1–3	
				Non-small cell lung carcinoma	NCT02106546 (Phase 3)	1–3	
				Solid tumors	NCT01193140 (Phase 2)	1–2	
				Testicular carcinoma	NCT02860819 (Phase 2)	1–2	
Talazoparib (BMN-673)	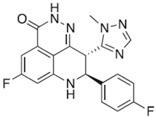	Amide motif enclosed in cyclic ring	IC_50_ = 1.2 nM	Breast carcinoma	NCT01945775 (Phase 3)	1–3	[85,86,99,100]
				Prostate carcinoma	NCT03148795 (Phase 2)	1–2	
				Ovarian carcinoma	NCT02326844 (Phase 2)	1–2	
				Endometrial carcinoma	NCT02127151 (Phase 2)	1–2	
				Acute Myeloid Leukemia	NCT02878785 (Phase 2)	1–2	
